# Efficient *Agrobacterium*-Mediated Transformation of Hybrid Poplar *Populus davidiana* Dode *× Populus bollena* Lauche

**DOI:** 10.3390/ijms14022515

**Published:** 2013-01-25

**Authors:** Xue Han, Shurong Ma, Xianghui Kong, Tetsuo Takano, Shenkui Liu

**Affiliations:** 1 Key Laboratory of Saline-alkali Vegetation Ecology Restoration in Oil Field (SAVER), Ministry of Education, Alkali Soil Natural Environmental Science Center (ASNESC), Northeast Forestry University, Harbin Hexing Road, Harbin 150040, China; E-Mails: hanxue_home@163.com (X.H.); msrbsc@163.com (S.M.); kxh29@126.com (X.K.); 2 Institute of Microbiology, Heilongjiang Academy of Sciences, Harbin 150010, China; 3 Asian Natural Environmental Science Center, University of Tokyo, Nishitokyo-shi, Tokyo 188-0002, Japan; E-Mail: takano@anesc.u-tokyo.ac.jp

**Keywords:** regeneration, transformation, leaf age, poplar, *Agrobacterium*

## Abstract

Poplar is a model organism for high *in vitro* regeneration in woody plants. We have chosen a hybrid poplar *Populus davidiana* Dode × *Populus bollena* Lauche. By optimizing the Murashige and Skoog medium with (0.3 mg/L) 6-benzylaminopurine and (0.08 mg/L) naphthaleneacetic acid, we have achieved the highest frequency (90%) for shoot regeneration from poplar leaves. It was also important to improve the transformation efficiency of poplar for genetic breeding and other applications. In this study, we found a significant improvement of the transformation frequency by controlling the leaf age. Transformation efficiency was enhanced by optimizing the *Agrobacterium* concentration (OD_600_ = 0.8–1.0) and an infection time (20–30 min). According to transmission electron microscopy observations, there were more *Agrobacterium* invasions in the 30-day-old leaf explants than in 60-day-old and 90-day-old explants. Using the green fluorescent protein (GFP) marker, the expression of MD–GFP fusion proteins in the leaf, shoot, and root of hybrid poplar *P. davidiana* Dode *× P. bollena* Lauche was visualized for confirmation of transgene integration. Southern and Northern blot analysis also showed the integration of T-DNA into the genome and gene expression of transgenic plants. Our results suggest that younger leaves had higher transformation efficiency (~30%) than older leaves (10%).

## 1. Introduction

Poplar has several exceptional qualities, such as a high capacity for vegetative propagation and a fast growth rate. It has been extensively used for the pulp and paper industry, reforestation of lowlands, and phytoremediation of contaminated soils. The micro-vegetative propagation and regeneration of poplar has been reported in recent decades. Different poplar genotypes have been used to perform regeneration experiments [1–10]. Growth and differentiation response is not only controlled by the cultural environment, but is also dependent upon the genotype [11]. *In vitro* regeneration has been attempted in several poplar species using different explants such as leaves [8,11–14], petioles [15], internodes [8,14], stems [5,16], roots [8,11], and shoot tips [17]. The leaf disc transformation method [18] has been widely used in plant genetic engineering.

Parsons *et al.* [19] first reported the genetic transformation of poplar. This technology has been applied to various *Populus* species to improve their transformation efficiency [20–28]. Several factors were systematically analyzed to improve transformation efficiency, including poplar genotype, *Agrobacterium tumefaciens* strain for transformation, bacterial concentration, acetosyringone (AS) [20,29–32], and different explants including leaf discs and stems [23,33].

It is important to improve the transformation efficiency of poplar for genetic breeding and other applications. For example, the improvement of the transformation efficiency is crucial to build the full-length (fl) cDNA overexpression (FOX) hunting system and T-DNA mutant system. FOX requires large numbers of dominant mutations that enable the comprehensive characterization of mutant phenotypes and the identification of functional genes. 15,000 transgenic lines were used for expressing *Arabidopsis* full-length (fl) cDNAs [34], and >23,000 independent *Arabidopsis* transgenic lines for expressing rice fl-cDNAs [35]; Nakamura *et al.* [36] generated approximately 12,000 FOX-rice lines. Busov *et al.* [37] also reported that from the 627 independent activation-tagged poplar lines, nine exhibited an obvious morphological phenotype that had never been seen among the thousands of transgenic poplars produced.

We have chosen a hybrid poplar *Populus davidiana* Dode *× P. bollena* Lauche to improve the transformation efficiency. *Populus* was planted in northeast China and has a well-characterized morphology with cold resistance features. We have optimized the Murashige and Skoog [38] medium with supplementary 6-Benzylaminopurine and Naphthaleneacetic acid to achieve a high regeneration efficiency of *Populus*. Subsequently, we attempted to maximize the transformation efficiency. Although several factors effecting transformation efficiency have been studied, there were no attention to explant age. In order to improve the transformation efficiency in this work, we optimized the conditions of the leaf explants age, as well as *Agrobacterium* concentration.

## 2. Results and Discussion

### 2.1. Optimization of Poplar Shoot Regeneration

**Figure 1:**
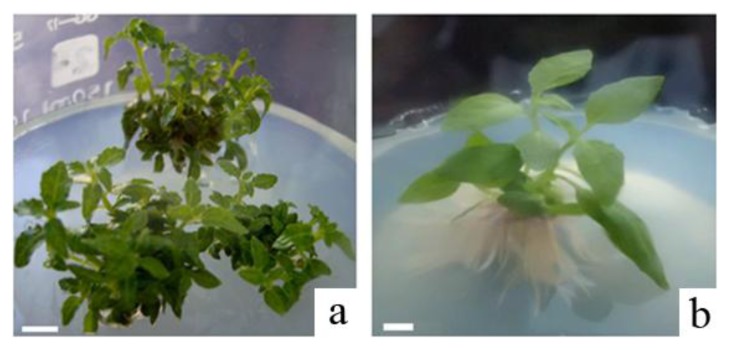
High-frequency plant regeneration of *Populus davidiana* Dode *× Populus bollena* Lauche. (**a**) Regenerated shoots from leaf explants; (**b**) Regenerated plantlet. Scale bar 1 cm.

Leaf explants of a hybrid poplar *Populus davidiana* Dode *× P. bollena* Lauche were cultured in media with 0.1~0.5 mg/L BA and 0.01~0.1 mg/L NAA. There were no explants regenerated for 0.1 mg/L BA and 0.01–0.03 mg/L NAA (Table 1). Low regeneration frequencies were induced using BA (0.1 mg/L) and NAA (0.05, 0.08, or 0.1 mg/L) and BA (0.5 mg/L) with NAA (0.01, 0.03, 0.05, 0.08, or 0.1 mg/L). Ninety percent of the explants regeneration shoots when BA (0.3 mg/L) and NAA (0.08 mg/L) were used. Regenerated shoots from leaf explants were shown in Figure 1a. Confalonieri *et al.* [20] reported the positive effect of BA and NAA on shoot regeneration in *Populus nigra*. To obtain shoot regeneration, leaf discs were cultivated in Petri dishes on MS medium with 0.5 mg/L BA and 0.2 mg/L NAA.

The rooting experiments showed that new root formation and growth were promoted by NAA at any concentration, and root regeneration was 100%. However, NAA at 0.25 mg/L was the optimal rooting concentration because the growth status of roots was long and polyrhizal (Figure 1b). After the hardening process, 90% of the poplar plantlets survived acclimatization.

Previously, various media were used to different genotypes and explants for shoot regeneration and rooting. IBA was used to induce roots for leaf of aspen and zeatin was used for shoot regeneration [24]. Adventitious shoots regenerated in a woody plant medium (WPM) supplemented with TDZ (Thidiazuron) from aspen stem explants [16]. Ferreira *et al.* [14] reported that adventitious shoot regeneration of *P. euphratica* from organogenic nodules of leaf explants was achieved within a range of concentrations of a-naphtalenacetic acid and 6-benzylaminopurine, at a fixed 2:1 ratio. Shoots of *Populus ciliata* Wall were regenerated at high frequencies from explants grown on Murashige and Skoog (MS) medium supplemented with 0.5 mg/L kinetin and 0.2 mg/L indole-3-acetic acid (IAA). Regenerated shoots developed roots in MS medium supplemented with 0.1 mg/L IAA [7].

### 2.2. Improvement of Genetic Transformation of Poplar

**Figure 2:**
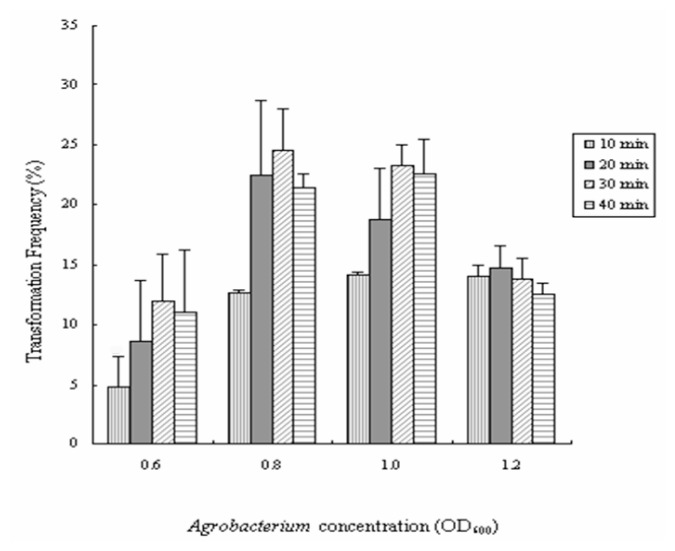
Frequency of transformation of *Populus davidiana* Dode × *Populus bollena* Lauche with different *Agrobacterium* concentrations and infection times.

The effect of *Agrobacterium* concentration on transformation frequency was shown in Figure 2. At OD_600_ = 0.6, a lower transformation frequency was observed. As OD_600_ ranging from 0.8 to 1.0, a higher transformation frequency was observed with little contamination. At OD_600_ = 1.2, the transformation frequency was also reduced because of a higher contamination frequency. At OD_600_ ranging from 0.8 to 1.0, the suitable incubation time of the explants in the bacterial suspension ranged from 20 min to 30 min (Figure 2). Previous study by Confalonieri *et al.* [20] optimized the production of transgenic plantlets from different *P. nigra* clones, *Agrobacterium* concentration (7 × 10^8^; 1.2 × 10^9^ cells/mL) were used. The results show that the optimal procedure involved dipping of leaf discs into a bacterial suspension (7 × 10^8^ cells/mL) for 20 min. Previous research has shown that the transformation efficiency increases in the presence of AS in the co-cultivation medium [32,39]. However, in the current experiment, the presence of AS during co-cultivation did not increase further the transformation efficiency (data not shown).

**Figure 3:**
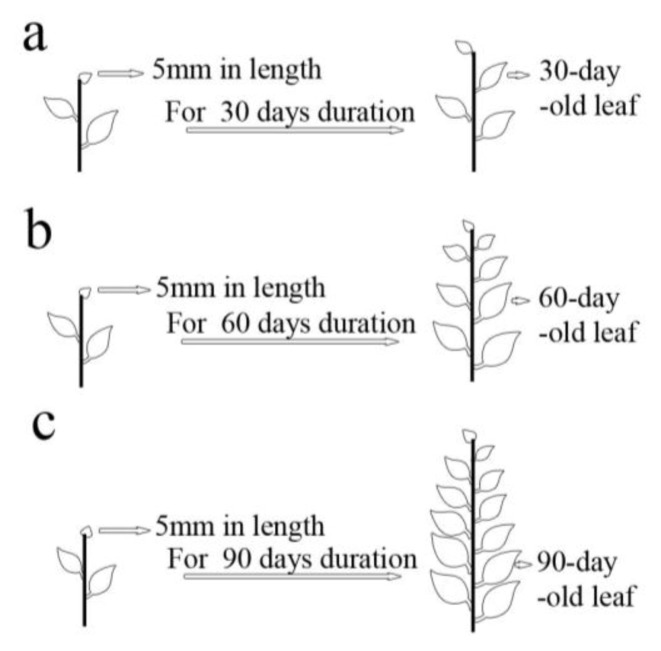
Leaf age definition diagram. Leaf age was defined as 30-, 60-, and 90-day-old growth from a 5 mm leaf in length.

**Figure 4:**
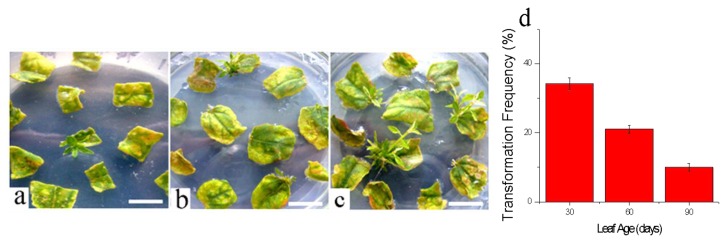
Transgenic shoot on regeneration-selection medium containing 50 mg/L Kan and 200 mg/L Cef. (**a**) 90-day-old leaf; (**b**) 60-day-old leaf; (**c**) 30-day-old leaf. Scale bar 1 cm. (**d**) Transformation percentage per leaf age from more than 100 samples.

In addition to *Agrobacterium* concentration, and infection time, the transformation frequency was also influenced by leaf age. We selected three different leaf age groups, 30-, 60-, and 90-day-old (Figure 3). The transformation frequency of the 30-day-old leaves was more than that of the 60-day-old and 90-day-old leaves (Figure 4). As the age of the sample leaf increased, the transformation frequency decreased (Figure 4d). The transformation frequency was 10% for the 90-day-old samples, while ~30% for the 30-day-old samples. Although several factors affecting transformation efficiency have been studied, there was no focus on explant age. A previous study by Smith and McCown [40] reported that juvenile sources of explant tissues have superior growth and regeneration potential compared with other sources. Civinova and Sladky [41] reported that juvenile explants have superior regeneration capacity and they are less lignified. Han *et al.* [22] reported that incompletely lignified explant sections of hybrid cottonwoods have also been efficiently transformed with *Agrobacterium*.

Different genotypes may respond differently to the optimal treatment conditions, and genetic transformation efficiency would be changed. According to published works, transformation frequency appeared to be different: 4%–6% for *Populus trichocarpa*, genotype Nisqually-1 [33], 16% for *Populus tremuloides* [27], and 20% for *Populus nigra var. italica* [26]. To improve its utility for functional genomics research, mutant library construction using the FOX hunting system was necessary for an efficient means for transformation and regeneration. Using hybrid poplar *Populus davidiana* Dode *× P. bollena* Lauche for transformation, the transformation frequency exceeded 30% by controlling the age of the leaf.

**Figure 5:**
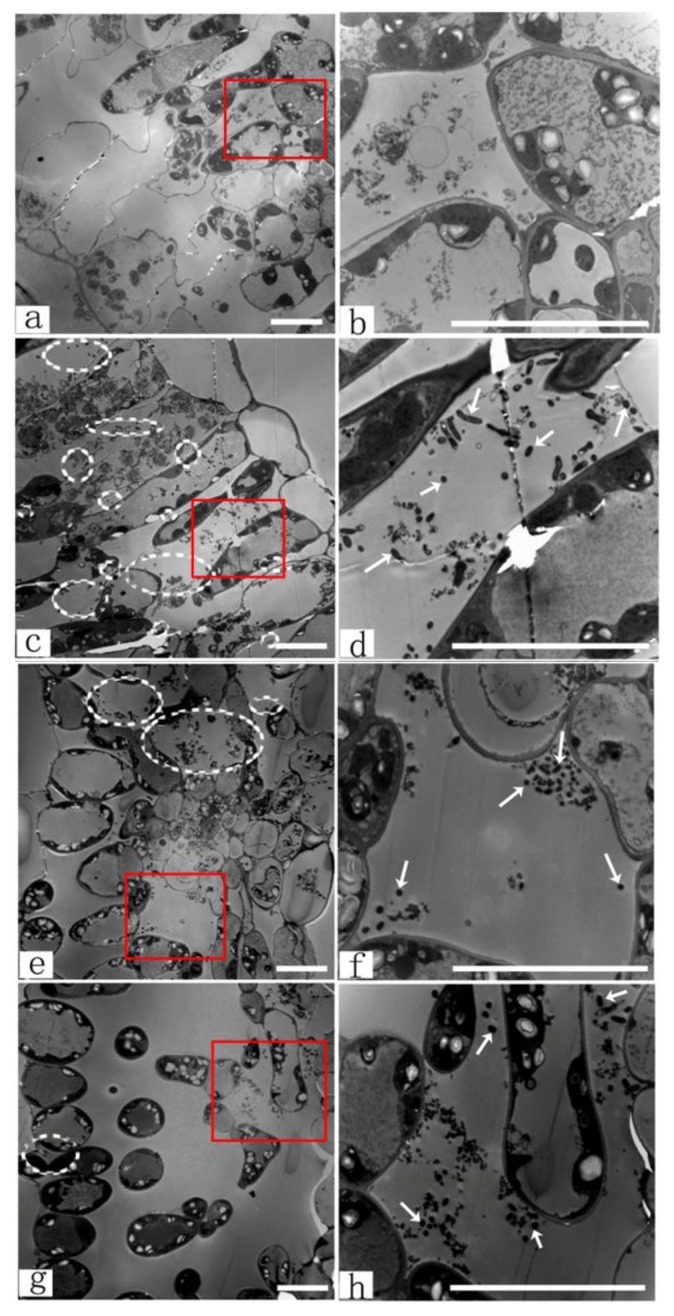
Transmission electron micrographs of the different leaf ages with *Agrobacterium* invasion in intercellular spaces. Leaf explants were infected with *Agrobacterium* at OD_600_ ranging from 0.8 to 1.0 and infected for 20 min to 30 min. The leaf explants were cut transversely. (**a**) and (**b**) Uninfected leaf as the control; (**c**) and (**d**) 30-day-old leaf explant; (**e**) and (**f**) 60-day-old leaf explant; (**g**) and (**h**) 90-day-old leaf explant. In **c**–**h**, *Agrobacterium* invasion into the intercellular space was indicated by white circles and arrows. The magnified images of the boxes in the left column (**a**, **c**, **e**, **g**) were placed in the right column (**b**, **d**, **f**, **h**), respectively. Scale bar 20 μm.

In order to understand the higher transformation ability in the younger leaf explants, the *Agrobacterium* invasion of various leaves were analyzed to confirm that leaf age was an important factor in *Agrobacterium-*mediated transformation. Figure 5a,b showed the magnified TEM image of the intercellular spaces with no *Agrobacterium* invasion. For 30-day-old leaf explants, some bacteria were scattered throughout the intercellular space (Figure 5c,d). For the 60-day-old leaf explants, the bacteria decreased slightly in number, and some of the intercellular space appeared empty (Figure 5e,f). For the 90-day-old leaf explants, almost all the intercellular space appeared clean and just a few bacteria were observed in the intercellular space (Figure 5g,h). These results demonstrated that there were *Agrobacterium-*mediated transgenic activities in the leaf explants, as well as the 30-day-old leaf explants contained more *Agrobacterium* than the 60-day-old and 90-day-old explants. The highest transformation efficiency was generated by the 30-day-old leaf explants (Figure 4d). The transformation frequencies were influenced by the amount of invasive bacteria.

Younger leaf explants may have a higher capability of shoot regeneration. However, in our TEM observations, the younger leaf explants had higher *Agrobacterium* infection. The invasion behavior of *Agrobacterium* in plant cells and their effects on genetic transformation is interesting. *Agrobacterium* invasion into plant cells can be affected by plant tissue age [42]. Younger poplar leaves were more susceptible to *Agrobacterium*-mediated transformation. As leaf age increased, the susceptibility to genetic transformation by *Agrobacterium* invasion decreased. A possible reason for the observed differential response of *Agrobacterium* invasion in different tissues was because of changes in the cell wall composition at different physiologic and developmental stages. As the plant cells mature, their cell wall composition changes so that *Agrobacterium*-binding sites were gradually lost [43].

### 2.3. Observations of Genetic Transformation

**Figure 6:**
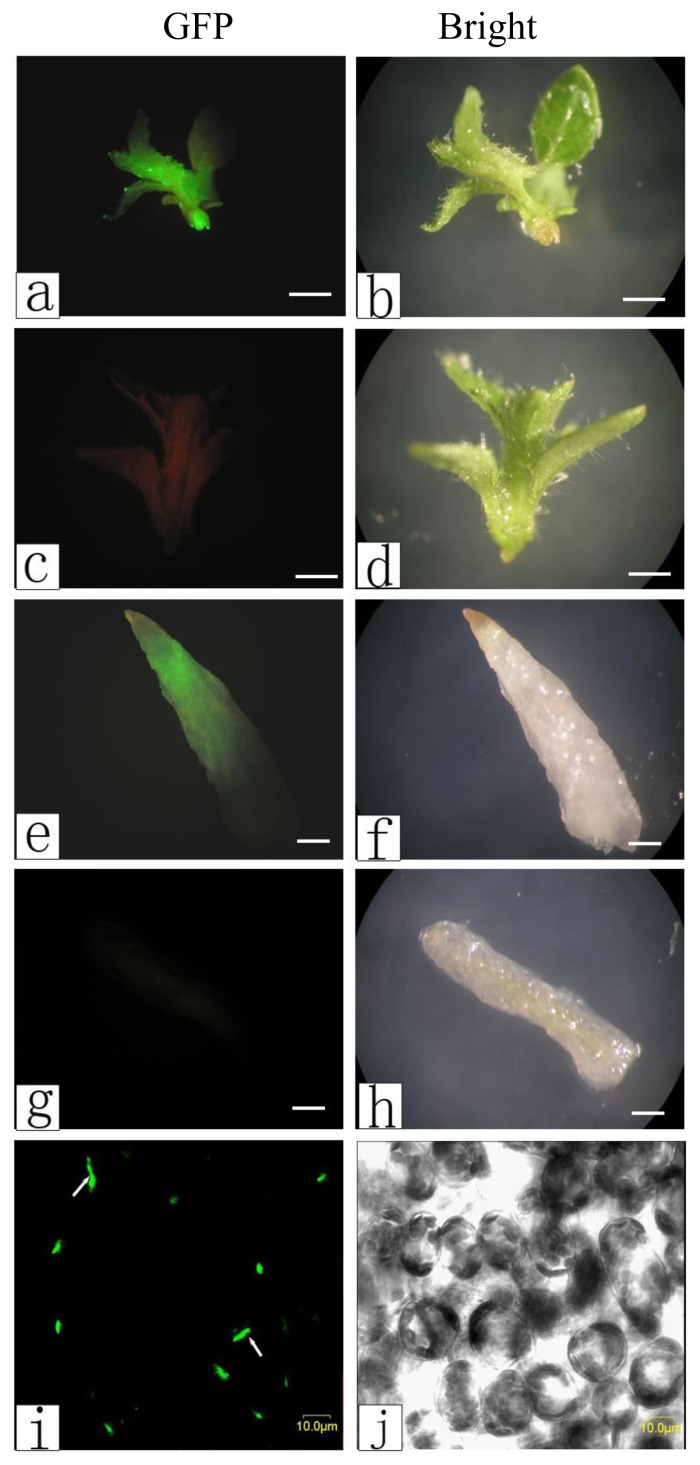
Expression of the MD–GFP fusion protein in transgenic poplar. Non-transgenic with no GFP signal in poplar shoots (**c**) and (**d**) and roots (**g**) and (**h**). GFP fluorescence in the shoots (**a**) and (**b**), roots (**e**) and (**f**), and leaf epidermis (**i**) and (**j**) of transgenics. Left column was the GFP fluorescence images and right column was the optical images. Scale bar (**a**–**h**) 0.2 cm; (**i**–**j**) 10 μm.

We observed the genetic transformation further using a GFP label and Southern and Northern blot analysis. A plant transformation vector designed to express the MD–GFP fusion protein was used to detect the GFP signals in transgenic poplar. There were no GFP signals in the non-transgenic plants, shoots (Figure 6c,d), or roots (Figure 6g,h). However, the GFP signals from the shoots (Figure 6a,b), roots (Figure 6e,f), and leaf epidermis (Figure 6i,j) of the transgenic plants were detected. These results demonstrated that the pBI121*-*MD-GFP plasmid was introduced into hybrid poplar *P. davidiana* Dode *× P. bollena* Lauche.

**Figure 7:**
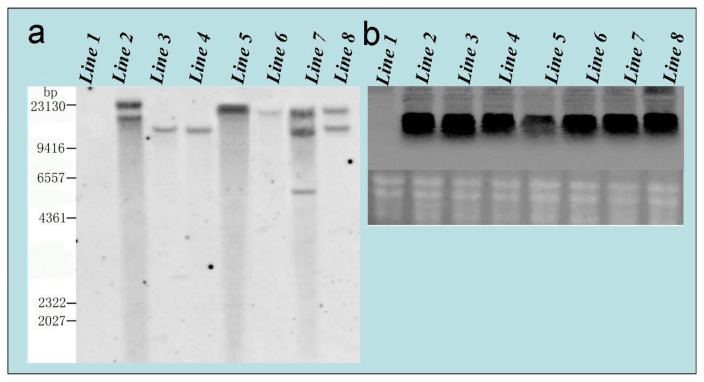
(**a**) Southern blot analysis of transgenic poplar. Poplar DNA was digested with Bam HI, electrophoresed, and probed with DIG-labeled MD–GFP gene. Molecular weight DNA markers were shown on the left. Lane 1: non-transgenic poplar (control), and Lanes 2~8: transgenic poplar lines; (**b**) Detection of the transgene by Northern blot analysis. Analysis of the exogenous MD–GFP expression of the transgenic lines by Northern blot. Lane 1: non-transgenic poplar (control), and Lanes 2~8: transgenic poplar lines.

Genomic DNA from seven of the different independent transgenic lines was subjected to Southern blot analysis. In line 1, non-specific DNA bands appeared as a result of the continuous distribution (line 1, Figure 7a), but all other lines contained the distinct bands. The banded MD–GFP gene was associated with high molecular weight invaded transgenic DNA(T-DNA), and produced the diverse copies as shown in line 2~8 (Figure 7a). The T-DNA appeared to be randomly integrated into the poplar genome.

For Northern blot analysis, all transgenic lines displayed a distinct band consistent with the predicted MD–GFP mRNA (lines 2~8, Figure 7b), whereas no hybridization signal was detected from the non-transgenic wild-type plant (line 1, Figure 7b). These results indicated that the MD–GFP gene was successfully integrated and expressed in the seven transgenic poplar lines 2~8 (Figure 7b).

## 3. Experimental Section

### 3.1. Plant Material, *Agrobacterium* Strain, Probe, and Plasmid

*Populus davidiana* Dode × *P. bollena* Lauche leaf explants were obtained randomly from *in vitro* shoot cultures and then used for regeneration and transformation. *Populus davidiana* Dode × *P. bollena* Lauche was hybrid by Xinjiang poplar as the male parent and aspen as female parent. The plasmid pBI121-MD-GFP and DIG-labeled MD gene probe were kindly provided by Prof. Che (Northeast Agricultural University) and used as a marker for plant transformation in our laboratory. The plasmid pBI121-MD-GFP with the CaMV 35S promoter was transferred into *Agrobacterium tumefaciens* EHA105 using electroporation method (Bio-Rad, Micropluser, Hercules, CA, USA).

### 3.2. Establishment of Regeneration System for Poplar

Murashige and Skoog (MS) medium [38] was supplemented with 6-benzylaminopurine (BA) at 0.1 mg/L to 0.5 mg/L and naphthaleneacetic acid (NAA) at 0.01 mg/L to 0.1 mg/L (Table 1). The leaves were cut into 1 cm × 1 cm blades and were cultured adaxial. The leaves were transferred to fresh regeneration medium at 10-day intervals. The regenerated shoots (3 cm to 5 cm in length) were excised and rooted on MS medium containing NAA at 0.1, 0.2, 0.25, 0.3, or 0.4 mg/L. All media included 3% (*w*/*v*) sucrose and 0.8% (*w*/*v*) agar. The pH of the media was adjusted to 5.8 after adding the plant growth regulation, and then autoclaved at 121 °C for 20 min.

After 1 to 2 months, the root formation was lignified, and the well-developed plantlets were removed from the media. The plantlets were gently washed under running tap water to remove the adhering media. For the hardening process, the plantlets were transferred into plastic cups containing a mixture of sterile compost. The regeneration experiments were designed with 10 Petri dishes for shoot regeneration and 10 triangular flasks for rooting per treatment, and each dish contained at least six leaf explants per treatment for shoot regeneration analysis, and each flask contained three shoots per treatment for rooting frequency analysis. Experiments were done in triplicate. The cultures were incubated at 25 °C ± 2 °C under a 16 h photoperiod (45 μmol m^−2^ s^−1^).

### 3.3. Plant Transformation

#### 3.3.1. Sample Cultivation

Leaves were cut into 0.5 cm × 0.5 cm blades, immersed in the bacterial suspension in Petri dishes, and inoculated for different durations. Leaf explants were then dried on sterile filter paper and cultured on the shoot regeneration medium without Kanamycin sulfate (Kan) and cefotaxime sodium (Cef) in the dark. After 48 h of co-cultivation, leaf explants were washed five to six times (20 min to 30 min each) in liquid co-cultivation medium containing 200 mg/L Cef, and then blotted dry on sterile filter paper. The explants were then transferred to shoot regeneration medium containing Kan and Cef for selection, and cultured at 25 °C ± 2 °C under a 16-h photoperiod (45 μmol m^−2^ s^−1^) for 4 to 6 weeks. The cultures were transferred to fresh selection medium at 15-day intervals until shoot differentiation. The shoots were then transferred to rooting medium containing Kan and Cef after regeneration selection. Approximately 100 leaf explants were used for each transformation experiment and replicated thrice. The transformation frequencies were calculated by dividing the number of Kan-resistant regenerated shoots with the number of infected explants.

#### 3.3.2. Effects of *Agrobacterium* Concentration and Infection Time

A single *Agrobacterium* colony was inoculated into 3 mL of liquid YEP (Yeast extract 10 g/L; Tryptone 10 g/L; NaCl 5 g/L) medium with 100 mg/L rifampicin (Rif) and 50 mg/L Kan, and then incubated at 28 °C for 24 h with constant agitation (175 rpm). An additional 30 mL of YEP was added and cultured overnight in the same culture conditions until OD_600_ = 1.2. After centrifuging at 5000 rpm for 5 min, the pellet was diluted to OD_600_ = 0.6, 0.8, 1.0, or 1.2 for transformation. The leaf explants were incubated in the bacterial suspension at 10, 20, 30, or 40 min. The experiment was repeated thrice with around 100~130 leaf explants per *Agrobacterium* treatment.

#### 3.3.3. Effects of Leaf Ages

Leaf explant age (leaf age) was timed since it was 5mm in length. The interval durations were 30-day-old leaf, 60-day-old leaf, or 90-day-old leaf (Figure 3). The experiment was repeated thrice with around 100 leaf explants per treatment. The leaf explants used for transformation were also examined under transmission electron microscopy (TEM).

### 3.4. Visualization of Genetic Transformation

#### 3.4.1. TEM Observations of *Agrobacterium* Infection

The leaf explants used for transformation were washed five times for 15 s in 0.2 M phosphate buffer (pH = 6.8) containing 0.9% (*w*/*v*) NaCl to remove the *Agrobacterium* that were not firmly attached after co-cultivation. The leaf explants were then sampled for TEM (H-7650, Hitachi, Kyushu, Japan). The method was carried out following the reference of Williams *et al.* [44].

#### 3.4.2. Green Fluorescent Protein Fluorescence Observations in Transgenic Poplar

The leaves, shoots, and roots were removed from the transgenic poplar and observed under the fluorescent microscope (Olympus, Nagano-ken, Japan) and using a laser-scanning confocal imaging system (Olympus Fluoview, FV500, Nagano-ken, Japan). The GFP fluorescent signals were detected at an emitting wavelength of 480 nm.

#### 3.4.3. Southern and Northern Blot Analysis

DNA and RNA were extracted from leaf samples of the non-transgenic poplar and 7 different independent transgenic lines poplar. Poplar genomic DNA was extracted using the procedure of Porebski *et al.* [45]. Total RNA were extracted using the RNeasy Mini Kit (Qiagen) according to the manufacturer’s instructions. For Southern analysis, a total of 20 μg genomic DNA was digested with restriction enzyme BamHI at 37 °C in a water bath overnight. The digested DNA fragments were then electrophoresed in 0.8% agarose gel. For Northern analysis, 10 μg total RNA was electrophoresed on a 1% formaldehyde agarose gel. DNA and RNA samples were hybridized with DIG-labeled MD gene probe, and samples blotted onto a Hybond N^+^- membrane (Amersham Pharmacia, Piscataway, NJ, USA). The membrane was hybridized and washed according to Sambrook *et al.* [46]. The signals were detected with CDP-Star detection reagent using a LAS-4000 plus image analyzer (Fuji Film, Tokyo, Japan).

## 4. Conclusions

A hybrid poplar *Populus davidiana* Dode *× Populus bollena* Lauche planted in northeast China with cold tolerance has been optimized for high regeneration efficiency (90%). Genetic transformation was improved in terms of the age of the leaf explants (30-day-old leaf), *Agrobacterium* concentration (OD_600_ = 0.8–1.0), and infection time (20–30 min). The transmission electron microscopy observations of *Agrobacterium* invasions in the leaf explants, the visualization of the green fluorescent protein marker, and Southern and Northern blot analyses supported that there was successful integration of T-DNA into the genome and gene expression of transgenic plants.

## Figures and Tables

**Table 1:** Effect of BA and NAA on shoot regeneration of leaf explants of *Populus davidiana* Dode *× Populus bollena* Lauche.

Medium	BA (mg/L)	NAA (mg/L)	Mean No. shoots formed per explant	Explant regeneration (%)
1	0.1	0.01	0	0
2	0.1	0.03	0	0
3	0.1	0.05	0.18 ± 0.51	14.4 ± 1.93
4	0.1	0.08	0.08 ± 0.02	5.56 ± 1.93
5	0.1	0.1	0.02 ± 0.02	2.22 ± 1.92
6	0.3	0.01	3.61 ± 0.27	76.7 ± 6.65
7	0.3	0.03	1.82 ± 0.27	60.0 ± 10.0
8	0.3	0.05	0.42 ± 0.15	17.8 ± 5.09
9	0.3	0.08	5.87 ± 0.49	90.0 ± 8.81
10	0.3	0.1	2.08 ± 0.34	55.6 ± 5.09
11	0.5	0.01	0.59 ± 0.17	14.4 ± 3.85
12	0.5	0.03	1.54 ± 0.68	16.7 ± 5.77
13	0.5	0.05	0.78 ± 0.31	40.0 ± 10.0
14	0.5	0.08	0.03 ± 0.03	3.33 ± 3.34
15	0.5	0.1	0.53 ± 0.12	5.56 ± 3.85

## Abbreviations

AS: Acetosyringone
BA: 6-Benzylaminopurine
Cef: Cefotaxime sodium
GFP: Green fluorescent protein
Kan: Kanamycin sulfate
MS: Murashige and Skoog
NAA: Naphthaleneacetic acid
Rif: Rifampicin
TEM: Transmission electron microscopy
FOX: Full-length cDNA overexpression
